# MicroRNAs involvement in fludarabine refractory chronic lymphocytic leukemia

**DOI:** 10.1186/1476-4598-9-123

**Published:** 2010-05-26

**Authors:** Manuela Ferracin, Barbara Zagatti, Lara Rizzotto, Francesco Cavazzini, Angelo Veronese, Maria Ciccone, Elena Saccenti, Laura Lupini, Andrea Grilli, Cristiano De Angeli, Massimo Negrini, Antonio Cuneo

**Affiliations:** 1 Dipartimento di Medicina Sperimentale e Diagnostica Università di Ferrara, Ferrara, Italy; 2 Dipartimento di Scienze Biomediche e Terapie Avanzate, Università di Ferrara, Ferrara, Italy

## Abstract

**Background:**

Fludarabine, is one of the most active single agents in the treatment of chronic lymphocytic leukemia (CLL). Over time, however, virtually all CLL patients become fludarabine-refractory. To elucidate whether microRNAs are involved in the development of fludarabine resistance, we analyzed the expression of 723 human miRNAs before and 5-days after fludarabine mono-therapy in 17 CLL patients which were classified as responder or refractory to fludarabine treatment based on NCI criteria.

**Results:**

By comparing the expression profiles of these two groups of patients, we identified a microRNA signature able to distinguish refractory from sensitive CLLs. The expression of some microRNAs was also able to predict fludarabine resistance of 12 independent CLL patients. Among the identified microRNAs, miR-148a, miR-222 and miR-21 exhibited a significantly higher expression in non-responder patients either before and after fludarabine treatment. After performing messenger RNA expression profile of the same patients, the activation of p53-responsive genes was detected in fludarabine responsive cases only, therefore suggesting a possible mechanism linked to microRNA deregulation in non-responder patients. Importantly, inhibition of miR-21 and miR-222 by anti-miRNA oligonucleotides induced a significant increase in caspase activity in fludarabine-treated p53-mutant MEG-01 cells, suggesting that miR-21 and miR-222 up-regulation may be involved in the establishment of fludarabine resistance.

**Conclusions:**

This is the first report that reveals the existence of a microRNA profile that differentiate refractory and sensitive CLLs, either before and after fludarabine mono-therapy. A p53 dysfunctional pathway emerged in refractory CLLs and could contribute in explaining the observed miRNA profile. Moreover, this work indicates that specific microRNAs can be used to predict fludarabine resistance and may potentially be used as therapeutic targets, therefore establishing an important starting point for future studies.

## Background

Chronic Lymphocytic Leukemia (CLL) is the most common hematologic neoplasia in the Western world and is characterized by a clonal expansion of CD5+ B-cells. The disease may have a long and indolent course, not requiring treatment for years, or it may rapidly progress. Initially most patients respond to purine nucleoside analogs, which today represent pivotal agents for first and second-line treatment [1]. However a significant fraction of patients do not respond or become resistant to fludarabine in the years [2] and prognosis for this group of patients is poor [3]. The molecular mechanisms underlying this process are not fully understood. It has been reported that p53 dysfunction, deriving from 17p13 deletion, predicts non-response to fludarabine therapy and poor prognosis [4,5]. Other genetic factors associated with poor prognosis, such as del(11q22.3) [6] and immunoglobulin (Ig) V(H) unmutated status [7], have been also associated with shorter progression-free survival after chemotherapy.

MicroRNAs (miRNAs) have been involved in the regulation of many physiological and pathological processes, through their wide action as post-transcriptional gene expression modulators [8,9]. In CLL, miR-15/16 were shown to be located within 13q14 deletion [10]; they were shown to act as tumor suppressor genes [11], possibly through the modulation of BCL2 [12] and various other target genes [13]. A germline mutation affecting miRNA maturation was detected in few cases of familial CLL as well as in the New Zealand Black (NZB) mouse strain, a mice developing a B lymphoproliferative disease that is a model for human CLL [14,15]. A set of miRNAs was able to distinguish CLLs from normal CD5+ B-cells [16] and 13 miRNAs associated to CLL prognostic groups were identified [17] through the identification of the molecular profiles of CLLs with IgVH mutated or unmutated status combined with ZAP70 levels. Likewise, miR-29c and miR-223 have been used to create a quantitative PCR-based score able to improve CLL patients stratification in terms of treatment free survival and overall survival, when combined with two other prognostic factors [18].

Recently, Zenz and colleagues found that fludarabine-refractory CLLs were characterized more frequently (53%) by lower levels of miR-34a than sensitive CLLs (9%) [19]. MiR-34a has been implicated in CLL response to DNA damage through a p53-mediated induction, while miR-106b has been linked to Itch inhibition, and consequent p73 activation, in deacetylase inhibitors treated CLLs [20].

Fludarabine is an active agent in CLL and, it is frequently used nowadays in combination regimens in first and second line treatment, especially in younger and fit patients. Fludarabine as single agent produces better response rates than chlorambucil and represents a reasonable choice as single agent in frail or elderly patients [21]. Because specific miRNAs may be involved in patients response to fludarabine treatment and in the development of fludarabine resistance, we designed this study aimed at identifying the miRNA expression profile associated with the response to fludarabine, used as a single agent, in the treatment of patients with CLL.

## Results

To investigate the possible involvement of miRNAs in fludarabine resistance, we analyzed the miRNA expression profile of 17 CLL patients, before and after 5 days of treatment with fludarabine as single agent. Based on NCI criteria, 9 of the patients exhibited a clinical response (CR), i.e. they attained either complete or partial response, while 8 patients were resistant to the treatment and were classified as non responders (NR). Characteristics of the enrolled patients are shown in Table 1. An additional blind cohort of 12 patients was recruited among patients who were treated with fludarabine (Table 1), to test the efficacy of specific miRNAs as response predictors.

**Table 1:** Characteristics of the patients

	Training set		Test set	
	**Not Responder group**	**Complete Responder group**	**Not Responder group**	**Complete Responder group**

	*n = 8*	*n = 9*	*n = 5*	*n = 7*
Male, n(%)	7 (87.5%)	6 (66.6%)	4 (80%)	2 (28.6%)
*Female, n(%)*	1 (12.5%)	3 (33.3%)	1 (20%)	5 (71.4%)
Median age at diagnosis, years(range)	67,5 (35-80)	66 (40-85)	68 (61-76)	60 (37-78)
11qdel, n(%)	0 (0%)	2 (22.2%)	0 (0%)	0 (0%)
17pdel, n(%)	2 (25%)	1 (11.1%)	3 (60%)	0 (0%)
Previous lines of therapy, n (%) (clorambucil)	8 (100%)	5 (55.5%)	4 (80%)	3 (42.9%)
Rai Stage, n (%)				
0-I	7 (87.5%)	9 (100%)	2 (40%)	4 (57.1%)
II-IV	1 (12.5%)	0 (0%)	3 (60%)	3 (42.9%)
Time to Treatment (months)	35.6 (0-75)	17.0 (0-56)	7.7 (0-20)	32.2 (10-75)

### Fludarabine induces a modulation of miRNA expression that exhibits a differential expression between responder and non-responder patients

**Figure 1:**
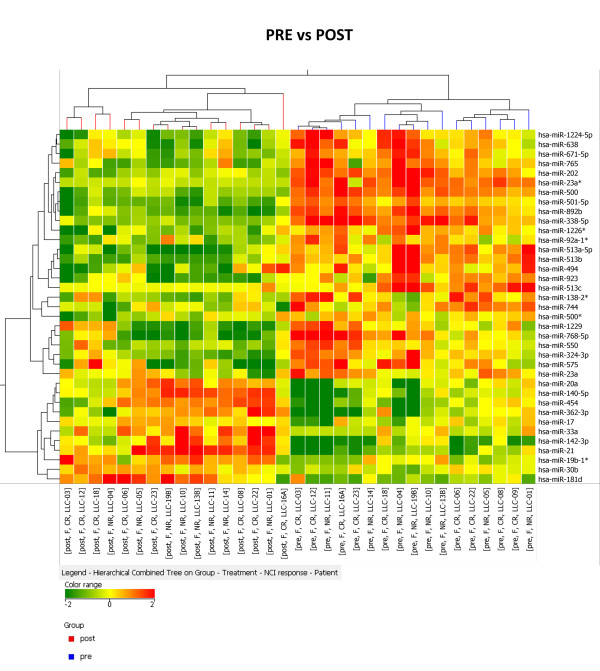
**MiRNAs modulated by fludarabine**. Cluster analysis of 17 CLL patients based on 37 miRNAs differentially expressed pre and post fludarabine treatment. Statistical analysis revealed that 37 miRNAs are modulated by the drug both in sensitive (CR) and refractory (NR) patients. The expression values of the genes represented on the heatmap correspond to the values normalized on miRNA mean expression across all samples. CLL pre treatment are colored in blue, post treatment in red.

We evaluated the miRNAs expression in 33 samples obtained from CLL patients before and after treatment, hereafter called pre and post, through the hybridization on a miRNA platform (Agilent technologies) able to assess the expression levels of 723 human miRNAs (MiRBASE release 10.1). First, we identified the miRNAs induced or repressed by fludarabine treatment by comparing post CLLs to the pre-treatment samples (fold change >1.5 and p-value < 0.05). We compared pre and post samples in the entire groups or in the NR and CR sub-groups separately, which revealed 97, 67 and 60 differentially expressed miRNAs, respectively. Among these, using Venn Diagram intersection, we identified 37 miRNAs that were differentially expressed either in the CR cohort and in the NR cohort of samples (Additional file 1: **Table S1 **and Additional file 2: **Table S2**). A hierarchical clustering algorithm, performed using the list of 37 miRNAs, correctly classified the pre versus post fludarabine samples (Figure 1). The complete list of miRNAs that were identified as differentially expressed either in the CR cohort or in the NR cohort of samples or both can be found in Additional file 2: **Table S2**.

**Figure 2:**
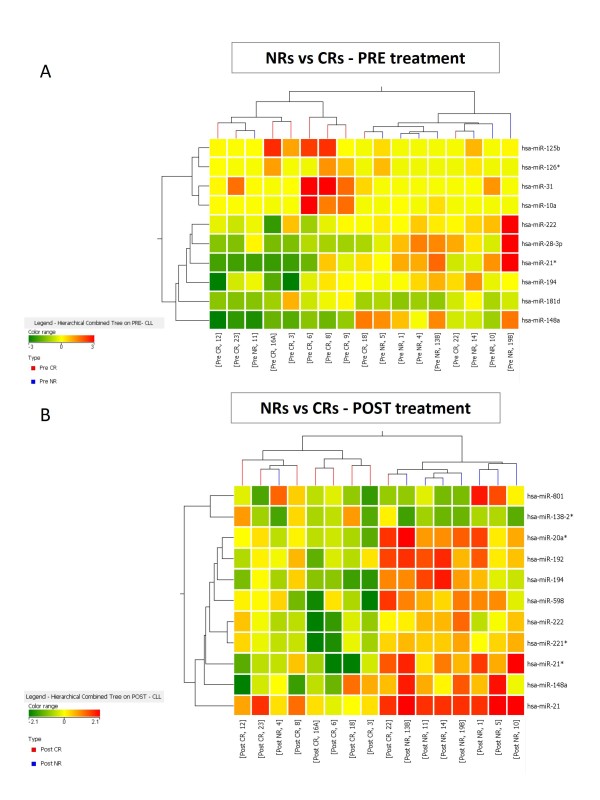
**Classification of CLL patients in accordance to miRNAs that differentiate fludarabine responders from not responders**. A) Eleven miRNAs are significantly (p < 0.05) modulated before fludarabine treatment between resistant and sensitive CLLs and were used for sample classification; cluster analysis revealed a good separation between the two classes. (B) Expression profile after fludarabine treatment; Ten miRNAs are differentially expressed (p < 0.05) in patients that will respond or not to chemotherapy; again, a good separation was achieved.

The post versus pre treatment analysis revealed that 37 miRNAs were similarly modulated by fludarabine in both NR and CR patients. This set of miRNAs indicated common mechanisms modulated by fludarabine, but could not reveal mechanisms underlying resistance to treatment. However, many additional miRNAs exhibited a different modulation in CRs versus NRs, which could be used to derive a miRNA signature associated with fludarabine resistance. To investigate this hypothesis, we searched for miRNAs whose expression was significantly different between these two cohorts of CLL patients. Analyses of pre and post treatment samples were performed separately. The lists of differentially expressed miRNAs were obtained by statistical analysis of microarray data (Table 2 and 3). The comparison of CR and NR patients led to the identification of 10 differentially expressed miRNAs in the pre-treatment setting (p < 0.05), listed in Table 2. This set of miRNAs was used to classify the patients by a clusterization algorithm. Eighty-two percent of patients were correctly classified by a cluster analysis (all but three: CLL 11, 18 and 22) (Figure 2A). The comparison of CRs and NRs in the post-treatment setting led to the identification of 11 miRNAs differentially expressed between resistant and sensitive patients (p < 0.05) (Table 3). Cluster analysis of these miRNAs was able to correctly classify 88% samples (all but two: CLL 4 and 22) (Figure 2B).

**Table 2:** MicroRNAs differentially expressed between refractory (NR) and sensitive (CR) patients before Fludarabine treatment

PRE Treatment Differentially expressed microRNAs							
microRNA	p-value	Fold change absolute	Regulation NR/CR	NR average expression	CR average expression	Chromosome	Accession
hsa-miR-31	0.0091	2.21	down	1.14	2.51	chr9	MIMAT0000089
hsa-miR-125b	0.0233	1.72	down	1.17	2.02	chr11	MIMAT0000423
hsa-miR-10a	0.0234	1.85	down	1.08	1.99	chr17	MIMAT0000253
hsa-miR-181d	0.0452	1.29	down	1.49	1.92	chr19	MIMAT0002821
hsa-miR-126*	0.0462	1.18	down	1.14	1.35	chr9	MIMAT0000444
hsa-miR-21*	0.0068	3.65	up	8.13	2.23	chr17	MIMAT0004494
hsa-miR-222	0.0096	2.12	up	70.59	33.31	chrX	MIMAT0000279
hsa-miR-28-3p	0.0201	3.38	up	5.04	1.49	chr3	MIMAT0004502
hsa-miR-194	0.0347	2.40	up	14.59	6.08	chr1	MIMAT0000460
hsa-miR-148a	0.0414	3.44	up	73.41	21.33	chr7	MIMAT0000243

**Table 3:** MicroRNAs differentially expressed between refractory (NR) and sensitive (CR) patients after Fludarabine treatment

POST Treatment Differentially expressed microRNAs							
microRNA	p-value	Fold change absolute	Regulation NR/CR	NR average expression	CR average expression	Chromosome	Accession
hsa-miR-138-2*	0.0142	1.91	down	1.39	2.65	chr16	MIMAT0004596
hsa-miR-21*	0.0168	3.11	up	9.60	3.09	chr17	MIMAT0004494
hsa-miR-148a	0.0188	2.77	up	119.81	43.27	chr7	MIMAT0000243
hsa-miR-221*	0.0212	1.96	up	9.75	4.97	chrX	MIMAT0004568
hsa-miR-21	0.0244	2.29	up	15,079.56	6,580.23	chr17	MIMAT0000076
hsa-miR-192	0.0266	1.95	up	35.14	18.04	chr11	MIMAT0000222
hsa-miR-194	0.0378	2.20	up	17.70	8.04	chr1	MIMAT0000460
hsa-miR-801	0.0408	1.81	up	44.94	24.83	chr1	MIMAT0004209
hsa-miR-598	0.0428	2.18	up	9.88	4.53	chr8	MIMAT0003266
hsa-miR-20a*	0.0448	1.99	up	37.27	18.77	chr13	MIMAT0004493
hsa-miR-222	0.0486	1.97	up	52.07	26.38	chrX	MIMAT0000279

We validated the microarray results for miR-21, miR-222 and miR-148a by quantitative reverse-transcription PCR (RT-qPCR) on the same group of 16 post samples, adding also 3 new samples. We chose these miRNAs considering the extent of their modulation and because they are differentially expressed both before and after the treatment. The expression level of every miRNA in every post CLL was assayed in triplicate and normalized on U6 RNA expression levels. Microarray results were confirmed by RT-qPCR in every group of CLLs (Additional file 3: **Figure S1**).

### Prediction of Fludarabine resistance in an independent cohort of patients

**Figure 3:**
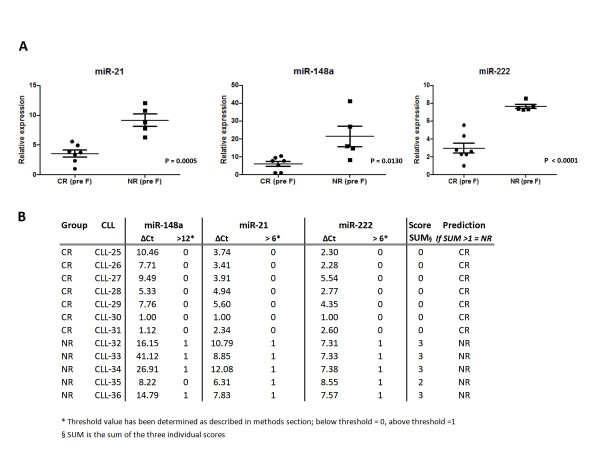
**Quantitative RT-PCR validation for miR-222, miR148a and miR-21 in independent CLL patients**. A) MiRNAs expression in a novel cohort of not responder (NR) and complete responder (CR) patients, before fludarabine treatment, quantified by using TaqMan reverse transcription qPCR. Each expression data is normalized on endogenous U6 RNA levels by 2^-ΔCt ^method. Each sample has been analyzed in triplicate. Data are displayed using vertical scatter plot (GraphPad v.5), bars represent means ± SEM. Two-tailed t-test was used to determine the p-values. B) Prediction of response to treatment in newly diagnosed CLL patients in accordance to a 3 miRNAs-based score. A threshold useful to predict response to therapy was established based on miRNA relative expression. Each patient with a final score >1 was classified as refractory.

To verify whether the three validated miRNAs (miR-21, miR-148a and miR-222) might be able to predict the efficacy of fludarabine treatment and might therefore become useful in directing patients therapy, we collected a new cohort of 12 patients (test set). These patients were treated by fludarabine as single agent because of disease progression. Samples were collected immediately before the treatment and the miRNA expression was assayed by RT-qPCR. In this new cohort, results were even more clear than in the microarray training set of samples (Figure 3A). Patients classified as NRs displayed a significantly (p < 0.05 at two-tailed t-test) increased expression of miR-21, miR-148a and miR-222 if compared to patients sensitive to treatment. Interestingly, we were also able to predict the response to treatment of newly diagnosed patients by using a score based on the combination of miR-21, 148a and 222 expression levels. Using this score based on quantitative expression of 3 miRNAs, prediction was 100% accurate (Figure 3B).

### Genes modulated by fludarabine in sensitive *-but not refractory- *CLLs are induced by p53

**Figure 4:**
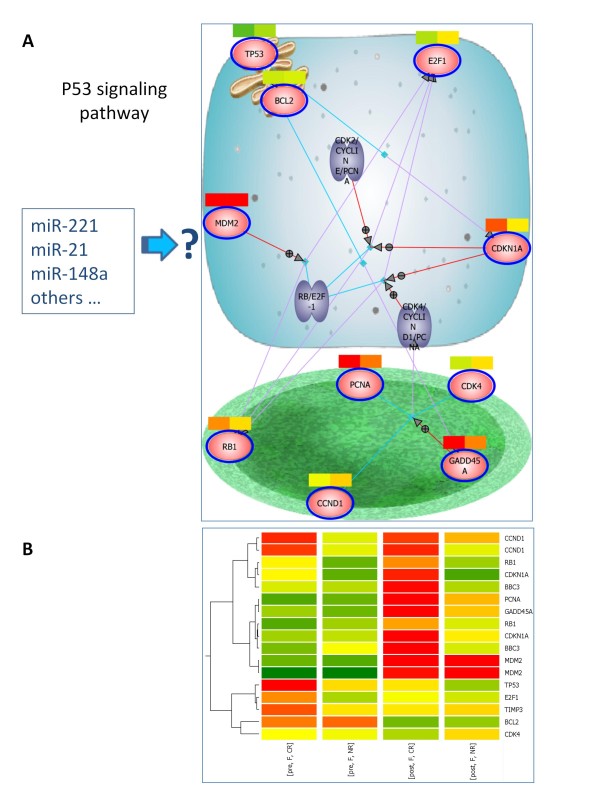
**Differential expression of p53-pathway genes in sensitive and refractory CLLs**. (A) Cellular representation of BioCarta p53 signaling pathway. Genes involved in p53 pathway were colored in accordance to microarray expression data for post CLLs normalized on matched pre samples. Average expression in CR patients is on the right part of the colorbar, NR patients on the left part. Genes like p21, GADD45A, PCNA are up-modulated only in sensitive CLLs. (B) Average expression of all the genes involved in p53 pathways (cell cycle control and apoptosis) in pre/post CR and pre/post NR groups. Puma/BBC3 gene is induced only in sensitive CLLs.

To better elucidate the molecular context responsible for the differential expression of miRNAs in NR versus CR patients, we performed a mRNA expression profile on a subgroup of the samples. Experiments data were obtained for pre and post fludarabine treatment of 14 patients (the same employed for miRNA analysis, except CLL-04, CLL-09 and CLL-16A, whose RNA was not available), 7 of them were CRs and 7 NRs. We hybridized the samples on a human mRNA platform (Agilent Technologies) able to assess the expression levels 41,000 human transcripts. We performed an analysis aimed at identifying the genes induced or repressed by the pharmacologic treatment, with a particular interest in genes modulated only in CRs or NRs. To this end, we normalized the expression of every gene in each post sample to the expression of the same gene in the matched pre sample. We identified the mRNAs that exhibited a significant differential modulation (p-value < 0.05) after fludarabine treatment, separately analyzing refractory and sensitive CLLs (data not shown). Then, we identified the most significant pathways modulated by fludarabine through a Pathway Enrichment analysis of the two gene lists. Interestingly, the p53 pathway emerged as the most significantly induced pathway in the cohort of CR patients (Table 4). For example, G1/S and G2/M DNA damage checkpoints, p53 stabilization and p53-dependent response, BH3-only proteins pathways were all significantly enriched in the CR cohort of CLL patients. Conversely, the same pathways were not enriched or were less significant in NR patients, indicating that NR CLLs had a dysfunctional p53 pathway. A pathway representative of p53 cell cycle control was generated, coloring the genes with the expression levels in CR and NR groups (Figure 4A), while the average expression level of all the genes involved in p53 pathway is graphically represented in Figure 4B.

**Table 4:** Pathways significantly enriched among the genes modulated by Fludarabine in sensitive and refractory CLLs

	Significant pathways in responder patients				Significant pathways in non responder patients			
Pathway	Number of Entities	Matched with Technology	Matched with EntityList	pValue	Number of Entities	Matched with Technology	Matched with EntityList	pValue
G1/S DNA Damage Checkpoints	11	2	2	5.40E-04	11	2	1	4.64E-02
Stabilization of p53	5	2	2	5.78E-04	5	2	1	5.08E-02
p53-Dependent G1 DNA Damage Response	7	2	2	5.78E-04	7	2	1	5.08E-02
p53-Dependent G1/S DNA damage checkpoint	7	2	2	5.78E-04	7	2	1	5.08E-02
BH3-only proteins associate with and inactivate anti-apoptotic BCL-2 members	9	3	2	1.70E-03				
p53 signaling pathway	14	11	3	1.98E-03				
atm signaling pathway	17	13	3	3.31E-03	17	13	2	4.28E-02
Class I PI3K signaling events	456	181	12	4.91E-03	456	181	10	1.43E-02
Aurora A signaling	117	43	5	5.49E-03				
hypoxia and p53 in the cardiovascular system	27	16	3	6.13E-03				
tumor suppressor arf inhibits ribosomal biogenesis	27	16	3	6.13E-03				
inhibition of cellular proliferation by gleevec	21	17	3	6.67E-03	21	17	2	5.93E-02
nfkb activation by nontypeable hemophilus influenzae	29	17	3	6.67E-03	29	17	2	5.93E-02
TRAIL signaling pathway	641	246	15	7.54E-03	641	246	15	3.24E-03
IL2 signaling events mediated by PI3K	110	33	5	7.73E-03				
Activation of BH3-only proteins	13	6	2	8.12E-03				
cell cycle: g2/m checkpoint	24	19	3	1.01E-02	24	19	2	8.48E-02
Signaling events mediated by HDAC Class III	44	21	3	1.22E-02	44	21	2	8.62E-02
repression of pain sensation by the transcriptional regulator dream	15	8	2	1.38E-02				
mapkinase signaling pathway	41	39	4	1.40E-02	41	39	3	7.84E-02
rna polymerase iii transcription	10	8	2	1.47E-02				
Signaling events regulated by Ret tyrosine kinase	131	40	4	1.61E-02				
Signaling by Aurora kinases	162	59	5	1.76E-02				
IL2-mediated signaling events	184	64	6	1.87E-02	184	64	5	6.96E-02
Role of Calcineurin-dependent NFAT signaling in lymphocytes	133	64	5	1.88E-02				
oxidative stress induced gene expression via nrf2	16	10	2	2.15E-02				
G(s)-alpha mediated events in glucagon signalling	5	1	1	2.33E-02				
Activation of PUMA and translocation to mitochondria	1	1	1	2.41E-02				
Activation of NOXA and translocation to mitochondria	1	1	1	2.41E-02				
G2/M DNA damage checkpoint	7	1	1	2.41E-02				
Intrinsic Pathway for Apoptosis	32	12	2	3.05E-02				
agrin in postsynaptic differentiation	23	13	2	3.55E-02				
cadmium induces dna synthesis and proliferation in macrophages	37	13	2	3.55E-02				
Plasma membrane estrogen receptor signaling	547	217	11	3.66E-02	547	217	9	8.14E-02
il-2 receptor beta chain in t cell activation	42	31	3	3.77E-02				
hypoxia-inducible factor in the cardivascular system	26	14	2	4.08E-02				
BMP receptor signaling	329	163	9	4.33E-02	329	163	8	4.54E-02
Class I PI3K signaling events mediated by Akt	160	55	4	4.35E-02				
rho-selective guanine exchange factor akap13 mediates stress fiber formation	11	2	1	4.60E-02	11	2	1	4.64E-02
FasL/CD95L signaling	6	2	1	4.75E-02				
alk in cardiac myocytes	20	15	2	4.93E-02	20	15	3	6.12E-03
role of mef2d in t-cell apoptosis	31	15	2	5.22E-02	31	15	4	4.38E-04
TNF receptor signaling pathway	495	213	11	7.61E-02	495	213	11	3.77E-02
integrin signaling pathway	28	21	2	9.19E-02	28	21	3	1.42E-02
FAS signaling pathway (CD95)					63	26	5	2.99E-03
endocytotic role of ndk phosphins and dynamin					22	6	2	7.76E-03
BCR signaling pathway					94	52	5	8.63E-03
Integrins in angiogenesis					107	38	4	1.18E-02
Alkaloid biosynthesis II					45	21	3	1.81E-02
angiotensin ii mediated activation of jnk pathway via pyk2 dependent signaling					39	24	3	1.81E-02
t cell receptor signaling pathway					44	23	3	1.81E-02
links between pyk2 and map kinases					39	24	3	2.02E-02
dicer pathway					4	1	1	2.57E-02
AndrogenReceptor					98	93	6	3.29E-02
Osteopontin-mediated events					48	13	2	3.62E-02
TCR signaling in naïve CD8+ T cells					188	72	5	3.66E-02
fas signaling pathway (cd95)					24	14	2	4.16E-02
Ceramide signaling pathway					90	55	5	4.48E-02

### Inhibition of miR-21 and miR-222 increases caspase activity after fludarabine treatment

**Figure 5:**
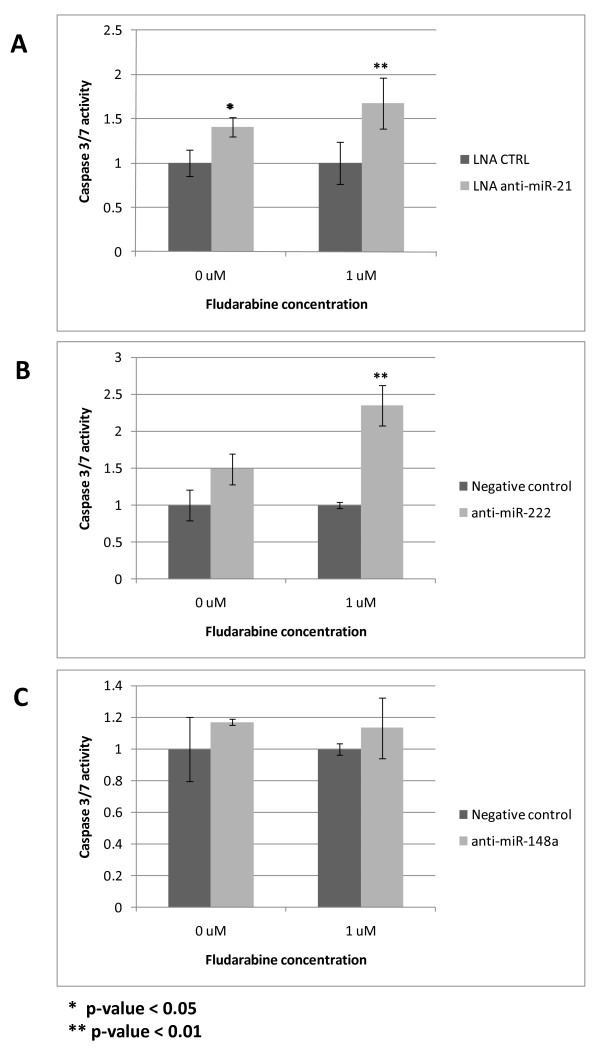
**Impact of anti-miRNAs on MEG-01 fludarabine sensitivity**. Caspase 3/7 activity after anti-miR-21, 222, 148a treatment of MEG-01 cells, in absence or presence of fludarabine (1 μM). LNA-anti-miR-21 and anti-miR-222, but not anti-miR-148a, were able to induce increase apoptosis in MEG-01 cells, which harbor a mutant p53 protein. The inhibition of miR-21 and miR-222 sensitizes the cells to fludarabine action, leading to an increased caspase activation when fludarabine 1 μM is added after 24 hours.

The above results suggest that an abnormal miRNA expression may be involved in the generation of fludarabine resistance. Among the various miRNAs differentially expressed between NR versus CR patients, miR-21, miR-222 and miR-148a emerged as significantly up-regulated in two different cohorts of refractory patients, both before and after fludarabine administration. MiR-21 has a well-established role in protection from apoptosis and drug-response modulation [22] and miR-222 is an important cell cycle regulator through its action on p27 [23,24]. We therefore investigated whether their inhibition could improve fludarabine cell death activity in the human MEG-01 cell model. We employed the MEG-01 cell line because it carries a 13q14 deletion affecting miR-15a/miR-16-1, typical of CLL, and a mutant TP53 gene. MiR-16 is indeed down-regulated (fold-change 2.3, p = 0.07) in pre-treatment fludarabine-refractory CLLs, and, as above indicated, NRs are defective in the p53 pathway. These cells may therefore mimic features which are responsible for resistance to fludarabine. We hypothesized that anti-miR-21, 148a and 222 could be implicated in fludarabine-induced apoptotic process. We therefore assayed the MEG-01 cells for Caspase 3/7 activation (Caspase 3/7 Glo assay, Promega); MEG-01 cells were treated for 24 hours with LNA anti-miR-21 or anti-miR-222 or anti-miR-148a followed by addition of fludarabine for additional 24 hours. A significant (p < 0.01) increase of caspase 3/7 activity was detected for miR-21 and miR-222 (Figure 5A, B) while no difference was observed for miR-148a (Figure 5C). We hereby concluded that the high miR-21 and miR-222 levels may be responsible for a weaker apoptotic effect of fludarabine in refractory patients, while the mechanism of action of miR-148a remains to be established.

## Discussion

We analyzed the expression profile of 29 CLL patients who were treated by fludarabine, a pivotal drug in the modern treatment of CLL [25,26]. To better elucidate the molecular mechanisms underlying the development of fludarabine resistance, we investigated the miRNA expression alterations of patients who received fludarabine as single agent, thus avoiding the potentially confounding elements that are inevitably present when fludarabine is used in combination with other drugs. Moreover, samples were obtained before and after first fludarabine treatment from the same patient, making it possible to directly evaluate the effect of the drug *in vivo*.

Our results revealed that many miRNAs are modulated by fludarabine treatment, as shown by the 37 miRNAs that are modulated following the treatment in both CR and NR patients, while 23 and 30 miRNAs were differentially modulated only in CRs or NRs, respectively, suggesting that these two cohorts of patients show signs of differences in the execution of molecular pathways modulated by fludarabine.

Indeed, in an effort to elucidate the mechanisms underlying the differential expression of these miRNAs, we analyzed the molecular pathways modulated by fludarabine in sensitive and refractory CLLs that emerged by mRNA expression analysis. The discovery that many p53 pathway genes (i.e. CDKN1A, BBC3/PUMA, PCNA, GADD45A) were activated in CRs but not in NRs provides further support to the notion that fludarabine treatment of patients with CLL induces a p53-dependent gene expression response [27] and that a defective p53 pathway in refractory CLLs, due to 17p deletions, p53 mutations and other p53-linked dysfunctions, is associated with fludarabine resistance [4,25,26,28]. It is therefore possible that the differential expression of miRNAs between CR versus NR patients may be affected by a defective p53 pathway. Interestingly, it has been reported that miR-221/222 cluster is repressed by p53 [29], implicating that a non-functional p53 pathway may lead to its up-regulation, as detected in NR patients. An example of an aberrantly expressed miRNA influenced by p53 in fludarabine-refractory CLL has been recently reported by Zenz and colleagues, who observed a higher frequency of fludarabine refractory CLLs in the group of low-expressing miR-34a, than in similar patients with high expression of this miRNA [30]. MiR-34a was shown to be a direct transcriptional target of p53 [31]. Although we did not find a significant differential expression level of miR-34a in NR compared with CR CLLs, our data concur with those of Zenz *et al.*, in fact 3 patients with the 17p deletion involved in this study displayed no miR-34a expression (data not shown).

By comparing the miRNA expression profile of sensitive and refractory CLLs, we have been able to identify expression signatures that could correctly classify more than 80% of the CR vs NR cases, either before or after treatment. Interestingly, after validation by quantitative PCR of the most significant miRNAs (miR-21, miR-222 and miR-148a) in an independent population, the prediction of response to therapy for pre-therapy samples was impressive: a predictive score based on miRNA expression levels reached an overall accuracy of 100%. This information may have important clinical implications because it might spare subsequent treatment in patients identified as resistant during disease progression.

MiRNAs aberrant expression may not only represent a phenomenon able to reveal an underlying molecular defect, such as p53 dysfunction, but it may also represent one of the possible mechanisms responsible for fludarabine resistance. In this study, we found that some miRNAs, namely miR-222, miR-148a and miR-21 were differentially expressed between NR and CR CLLs both before and after fludarabine treatment and were all up-regulated in the NR group. These findings, not previously described in CLL, appears to be truly significant.

Indeed, miR-222 can target the cell growth suppressive cyclin-dependent kinase inhibitors p27 and p57 and the c-KIT receptor (source: Tarbase) thus promoting cell cycle progression [23,32]. Among the recently validated targets there are the BH3-only pro-apoptotic protein BMF [33], the cell adhesion molecule ICAM-1 [34], matrix metalloproteinase 1 (MMP1) and manganese superoxide dismutase 2 (SOD2), which are both involved in tongue carcinoma metastasis promotion [35] and whose expression is frequently altered in cancer. An higher expression of miR-222 has been associated with poor survival in pancreatic cancer [36] and the enforced expression of miR-221/222 improved the growth of prostate carcinoma xenografts in mice [37]. Moreover, anti-sense inhibition of miR-221 could increase susceptibility to gemcitabine [38].

MiR-21 up-regulation appears to be an important mechanism of malignant transformation, since it was found up-regulated in several types of neoplasia (see Spizzo et al. for a list [39]); it accounts, among the known validated targets, numerous tumors suppressor genes, such as TPM1, PDCD4, maspin (SERPINB3) and PTEN (source: Tarbase). New interesting targets of miR-21 were recently identified through the validation of proteomic results [40]. It was also correlated with poor prognosis in tongue squamous cell carcinoma [41], colon [42], breast [43] and pancreatic cancer [44]. Interestingly, the use of the anti-miR-21 AMOs increases susceptibility of colangiocarcinoma cells to gemcitabine [45] and elicit a pro-apoptotic response in glioblastoma and breast cancer cells [46,47].

Here, we report that inhibition of miR-222 and miR-21 in the MEG-01 human cells using an anti-miRNA oligonucleotide increases susceptibility to fludarabine, as shown by the significant increase in caspase activity. Our findings suggest a direct role of miR-222 and miR-21 in conferring resistance to fludarabine chemotherapy independently from the p53 pathway, which is dysfunctional in this human cell line [48].

Less clear is the role of miR-148a. MiR-148a is up-regulated in NR CLL patients either before and after fludarabine treatment and it was identified also as resistance predictor in pre CLLs. However, its mechanism of action is not clear, as only two gene targets are presently experimentally validated. One is the isoform 1 of DNA methyltransferase 3b (DNMT3b1) [49] and the other is the pregnane X receptor gene [50] whose repression, in turn, reduces the expression of CYP3A4 enzyme. Our assay revealed that its mechanism of action may not be linked to apoptosis inhibition in resistant patients. Therefore, its role in fludarabine resistance remains unknown.

## Conclusions

This study revealed that a characteristic miRNA expression profile, as assessed before and soon after start of treatment, can discriminate between fludarabine sensitive and fludarabine resistant CLLs. We confirm the importance of a defective p53 pathway in conferring fludarabine resistance. This mechanism may be relevant in affecting miRNA expression; however, we suggest that an aberrant miRNA expression, i.e. up-regulation of miR-21, miR-148a and miR-222 in NRs, may be important in establishing resistance independently from p53, protecting cells from apoptosis. Indeed, inhibition of miR-21 and miR-222 may increase sensitivity to fludarabine in vitro in a p53-mutant cell line. The differential expression of a small number of miRNAs can have an effect on the capability to respond to fludarabine in CLL patients and can be used for response prediction. Therefore our data may help identify those elderly patients unlikely to respond to fludarabine used as single agent and may assist in the definition of the induction treatment in younger patients, given the availability of non cross-resistant drugs, such as bendamustine [51]. This information may have an impact in the development of personalized therapeutic approaches.

## Methods

### Patients

Seventeen patients (training set) plus 12 patients (test set) followed at our centre and treated with fludarabine as single agent (25 mg/sqm/day for 5 consecutive days for six courses given at 28 day intervals) were enrolled in this study after obtaining informed consent. The training set patients received fludarabine as first line treatment in 4 cases and as second line treatment after chlorambucil in 13 cases. All the patients had typical morphologic features (i.e. majority of small lymphocytes with clumped chromatin and inconspicuous nucleoli) and a high immunophenotypic score (i.e. CD5/CD19+, CD23+, CD22 weak positive, surface immunoglobulin weak+, FMC7-), consistent with an unequivocal diagnosis of CLL. Cytogenetic and fluorescence in situ hybridization studies were performed as previously described [52] as part of the diagnostic workup before treatment. Response to treatment was defined according to NCI criteria [53] and patients were divided in two groups, i.e. those attaining a clinical response (CR) including partial and complete responders and those who did not respond to treatment (NR).

For miRNA/mRNA expression studies, peripheral blood (PB) lymphocytes were separated to a >97% as previously described [54]. In each patient of the training cohort, cells were collected one day before the start of the first course of fludarabine and on day 5 of treatment. In the 12 patients constituting the test set, cells were collected one day before treatment start. RNA was isolated using Trizol LS Reagent (Invitrogen) according to manufacturer's instructions. Sample quality was assessed by Agilent 2100 Bioanalyzer (Agilent Technologies).

### Human miRNA/mRNA expression detection

MiRNA expression was investigated using the Agilent Human miRNA microarray v.2 (#G4470B, Agilent Technologies). This microarray consists of 60-mer DNA probes synthesized in situ and contains 15,000 features which represent 723 human miRNAs, sourced from the Sanger miRBASE database (Release 10.1). mRNA expression was detected using thee Agilent whole human genome oligo microarray (#G4112F, Agilent Technologies). This microarray consists of 60-mer DNA probes synthesized in situ, which represent 41,000 unique human transcripts. About 500 ngs of total RNA were employed in each experiment. RNA labeling and hybridization were performed in accordance to manufacturer's indications. Agilent scanner and the Feature Extraction 10.5 software (Agilent Technologies) were used to obtain the microarray raw-data.

### Microarray data analysis

Microarray results were analyzed by using the GeneSpring GX 10 software (Agilent Technologies). Data transformation was applied to set all the negative raw values at 1.0, followed by a Quantile normalization and a log2 transformation. Filters on gene expression were used to keep only the miRNAs/mRNA expressed in at least one sample (flagged as P). Then, samples were grouped in accordance to treatment (pre and post fludarabine) and response to treatment (NR and CR). Differentially expressed genes were selected as having a n-fold expression difference between their geometrical mean among the groups of interest and a statistically significant p-value (<0.05) by Welch t-test statistic. For gene expression statistical analysis, the Benjamini and Hoechberg correction for false positives reduction was used. Differentially expressed genes were employed in Cluster Analysis, using the Manhattan correlation as a measure of similarity and the complete linkage rule for genes and samples clusterization. The identification of mRNAs modulated after fludarabine, in matched samples, was done using the filter on 0-10th percentile in at least 75% of patients, for the miRNAs down-modulated by treatment, and a filter on 90-100th percentile in at least 75% of patients, for the miRNAs up-modulated by treatment. All Microarray data were submitted to ArrayExpress, accession number to be received.

### Pathway Analysis

The Pathway Enrichment Analysis was performed using GeneSpring GX tools, based on KEGG, BioCarta, Biopax, Cellmap and NCI-Nature databases.

### Quantitative RT-PCR

Quantitative Real time RT-PCR (qRT-PCR). Mature miRNAs expression was evaluated by Taqman MiRNA assays (Applied Biosystem) specific for miR-21, miR-222, miR-148a and RNU6B as reference gene according to the manufacturer's protocol. Briefly, 5 ng of total RNA was reverse transcribed using the specific looped primer; real time quantitative PCR was conducted using the standard Taqman MiRNA assay protocol on a Biorad-Chromo4 thermal cycler. Each sample was analyzed in triplicate. The level of miRNA was measured using Ct (threshold cycle). The amount of target, normalized on U6 RNA amount, was calculated using 2^-ΔCt ^(Comparative Ct) method as implemented by Biorad Genex macro for excel program. Significance in qRT-PCR results was determined by t-test. Box plot graphs were created using Microsoft Excel program.

### Calculation of a predictive score

For miR-21, miR-222 and miR-148a, a CR vs NR threshold value was determined by calculating and selecting the first integer number above the 98th percentile of expression values (as described in quantitative RT-PCR method) distribution in CR group. For each miRNA, a score of 0 was assigned when expression was below the threshold and a score of 1 when above. The sum of these scores for the three miRNAs was used to assign patients to one of two classes (CR or NR): for scores 0 to 1, the patient was predicted as CR; for scores 2 to 3, the patient was predicted as NR.

### Cell lines and transfection

MEG01 (ATCC number CRL-2021), cell line was cultured with RPMI-1640 medium with 10% fetal bovine serum and 0.1% gentamicin. LNA knockdown probe anti-miR-2 and Scramble-miR control were from Exiqon, Negative control, anti-miR-148a and anti-miR-222 were from Ambion. Transfection of oligos was carried out with lipofectamine 2000 (Invitrogen) and Optimem reduced serum medium (Invitrogen) in accordance with manufacturer's procedure.

### Caspase 3/7 assays

Caspase 3/7 activity assay was performed on MEG01 cell line. Cells were cultured in 96-well plates the day before LNA-anti-miR-21, anti-miR-222, anti-miR-148a and control transfections (50 nmol). After 24 hours from transfection, cells were treated with fludarabine at the concentrations of 0 and 1 μM for additional 24 hours. The assay was performed in accordance with manufacturer's protocol (Promega, Caspase-Glo 3/7 assay). Each experiment was performed in triplicate.

## List of abbreviations used

CLL: Chronic Lymphocytic Leukemia; miR or miRNA: microRNA; LNA: Locked nucleic acid; RT-qPCR: reverse transcription-quantitative polymerase chain reaction; CR: clinical response (complete + partial responder); NR: not responder.

## Competing interests

The authors declare that they have no competing interests.

## Authors' contributions

MF performed the research, analyzed data and wrote the paper, BZ performed microarray experiments, LR, FC, MC and CDA contributed patient samples and data, AV, LL and ES performed the research, AG performed bioinformatics analysis, AC and MN designed the research and wrote the paper. All authors critically reviewed and edited the paper.

## Supplementary Material

Additional file 1 **Table S1**. MicroRNAs modulated by fludarabine in sensitive and refractory CLL patients Click here for file

Additional file 2 **Table S2**. MicroRNAs modulated by fludarabine in either sensitive or refractory CLL patients Click here for file

Additional file 3 **Figure S1**. Quantitative RT-PCR validation for miR-222, miR148a and miR-21 in CLL patients. MiRNAs expression in Not Responder (NR) and Complete Responder (CR) patients, after fludarabine treatment, was quantified using TaqMan Real-time RT-PCR. Every expression data was normalized on endogenous U6 RNA levels by 2^-ΔCt ^method. Each sample was analyzed in triplicate. Data are displayed using vertical scatter plot (GraphPad v.5), bars represent means ± SEM. Two-tailed t-test was used to determine the p-values. Click here for file
