# Supplementary figures and images for: MicroRNAs involvement in fludarabine refractory chronic lymphocytic leukemia
Source: Mol Cancer. 2010 May 26;9:123. doi: 10.1186/1476-4598-9-123 (PMC2892453; doi:10.1186/1476-4598-9-123)

## Slide 1
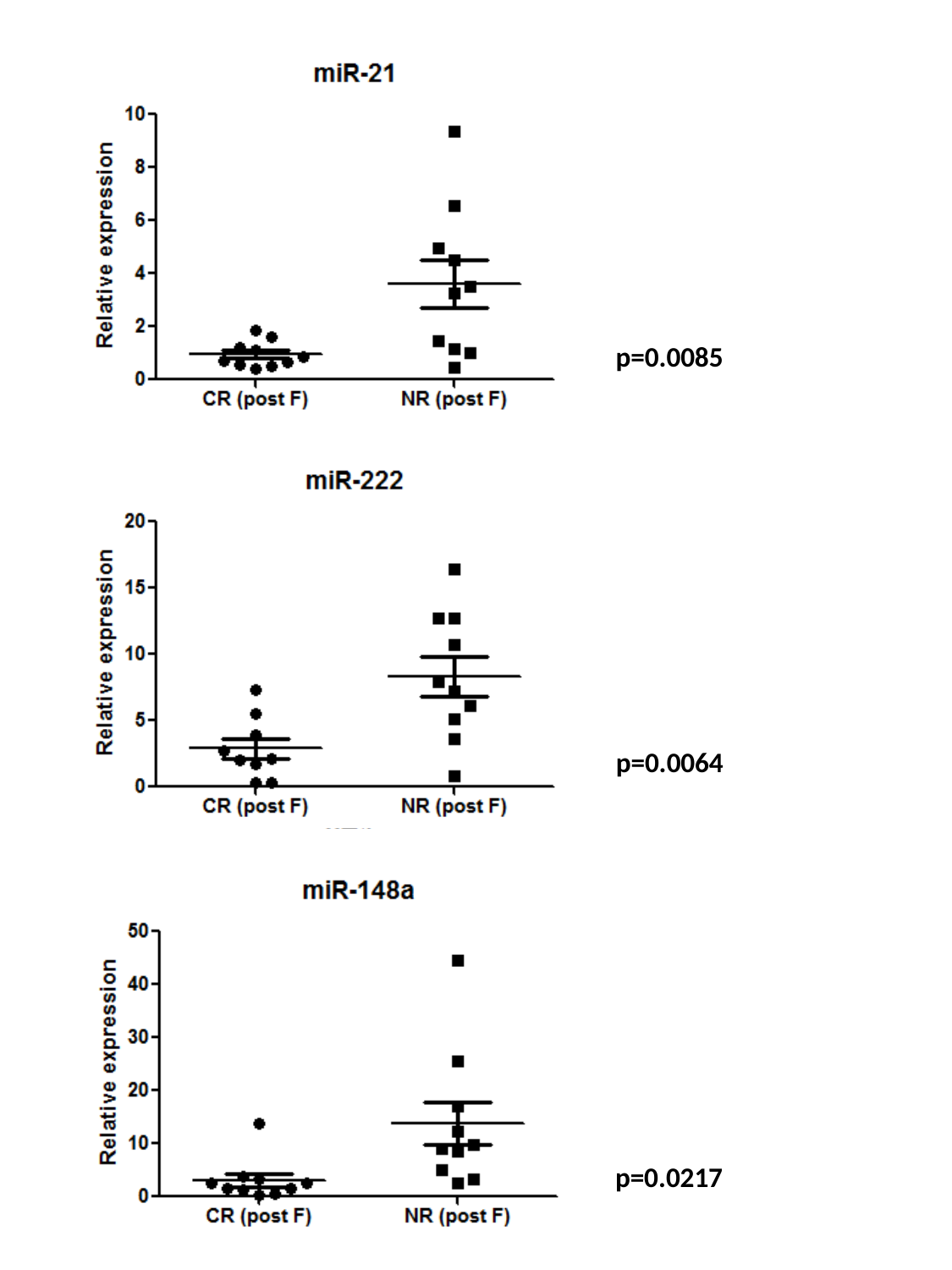

p=0.0085
p=0.0064
p=0.0217

Supplement: Additional file 3 — Figure S1. Quantitative RT-PCR validation for miR-222, miR148a and miR-21 in CLL patients. MiRNAs expression in Not Responder (NR) and Complete Responder (CR) patients, after fludarabine treatment, was quantified using TaqMan Real-time RT-PCR. Every expression data was normalized on endogenous U6 RNA levels by 2-ΔCt method. Each sample was analyzed in triplicate. Data are displayed using vertical scatter plot (GraphPad v.5), bars represent means ± SEM. Two-tailed t-test was used to determine the p-values. [file 1476-4598-9-123-S3.PPT]
